# The effects of polarization characteristics on visual fatigue: an empirical study based on subjective and objective indicators

**DOI:** 10.3389/fnins.2025.1715236

**Published:** 2025-11-27

**Authors:** Feng Si, Shuang Miao, Yi Yang, Shun Li, Guoli Liu, Wei Cao, Ruichen Zhang, Zihan Yang, Haibo Yang, Kaixuan Wang, Yunhong Zhang

**Affiliations:** 1 Key Laboratory of Human Factors and Ergonomics, State Administration for Market Regulation, China National Institute of Standardization, Beijing, China; 2 BOE Technology Group Co., Ltd., Beijing, China; 3 Faculty of Psychology, Tianjin Normal University, Tianjin, China

**Keywords:** polarized light, visual fatigue, subjective alertness, blink rate, EEG spectral features, alpha centroid frequency, integrated evaluation

## Abstract

**Introduction:**

This study aimed to examine the impact of circularly polarized versus linearly polarized displays on visual health, with a focus on visual fatigue during prolonged screen use.

**Methods:**

Eighteen healthy adults participated in a within-subject design. Under controlled illumination and display parameters, participants performed a 40-min standardized visual task. Electroencephalography (EEG) and Vertical electrooculography (VEOG) were recorded concurrently, while subjective questionnaires were administered to assess visual fatigue and alertness.

**Results:**

Compared with linearly polarized displays, circularly polarized displays were associated with significantly smaller increases in subjective visual fatigue and smaller declines in alertness (ps < 0.05). Blink rate remained stable under circular polarization but increased significantly under linear polarization and higher than in circular polarization (*p* < 0.05). EEG analyses revealed that circular polarization preserved stable neural activity, whereas linear polarization elicited a significant increase in the (θ + α)/β ratio and a significant decrease in alpha centroid frequency (ps < 0.05), indicating reduced cortical activation and slowed neural processing.

**Conclusion:**

Circularly polarized displays, by more closely resembling the optical properties of natural light, can effectively mitigate visual fatigue induced by prolonged screen viewing. These findings provide both theoretical insights and empirical evidence to inform the development of healthier display technologies and ergonomics-related standards.

## Introduction

With the rapid development of electronic display technologies, the use of display devices, such as computer monitors, tablets, and smartphones, has steadily increased in work, entertainment and study. This trend has been accompanied by a marked rise in visual fatigue, dry eye, and other ocular discomfort symptoms. Prolonged exposure to screens can lead to visual display terminal (VDT) syndrome, which has become a widely recognized public health concern (Kahal et al., 2025). VDT syndrome not only exacerbates visual fatigue but may also impair attention, reduce productivity, and provoke long-term ocular health risks, highlighting the need for effective mitigation strategies.

Light polarization refers to the orientation and temporal variation of the electric field vector in an electromagnetic wave, commonly manifested as linear, circular, elliptical, or unpolarized light. Linear polarized light vibrates in a fixed direction, whereas circularly polarized light is generated by the superposition of two orthogonal components with a 90° phase difference, producing a rotational trajectory in the plane (Hecht, 2017). Conventional liquid crystal displays (LCDs) primarily emit linearly polarized light, which may exert directional stimulation on retinal ganglion cells and increase visual load (Dodt and Kuba, 1990; Hochheimer and Kues, 1982). In contrast, circularly polarized light more closely approximates the isotropic properties of natural light, providing gentler stimulation and helping maintain visual comfort and stability (Yan et al., 2009).

The optical and physiological characteristics of the human visual system provide a theoretical basis for the superiority of circular polarization. Human ocular tissues such as the cornea and crystalline lens exhibit birefringence and partial depolarization effects due to their layered collagen structures and refractive interfaces (Bour, 1991; Bueno and Campbell, 2003). When linearly polarized light passes through these birefringent media, its polarization direction and phase may vary with incidence angle, leading to luminance modulation and visual instability on the retina. In contrast, circularly polarized light, which lacks a fixed vibration direction, is less affected by the orientation-dependent birefringence of ocular media, thereby maintaining more uniform transmission and visual consistency. Moreover, the human macula exhibits anisotropic absorption of linearly polarized light due to the radially arranged xanthophyll pigments in the Henle fiber layer, which gives rise to the Haidinger’s brush phenomenon (Haidinger, 1844; Le Floch et al., 2010). This effect can induce polarization-dependent luminance variations and perceptual fatigue. Circular polarization effectively avoids this orientation-selective absorption, resulting in reduced visual artifacts and a more stable viewing experience. Based on these theoretical considerations, circular polarization provides better visual comfort and optical stability compared with linear polarization.

A substantial amount of empirical research supports this hypothesis. Shixian et al. (2008) measured blink rate via electrooculography (EOG) and found a significant post-task increase in the linear polarization group, while no significant change was observed in the circular polarization group, suggesting that linear polarization may induce more severe symptoms of dry eye and visual fatigue. Xiaolin et al. (2009) compared the effects of circularly and linearly polarized LCD TVs in use, reporting that circular polarization significantly mitigated graded visual acuity decline and increase of blink rate during prolonged viewing, particularly in children. Li et al. (2016) recorded eye movement parameters and found that circularly polarized LCDs alleviated visual fatigue more effectively than linear ones during extended viewing sessions. Zhang et al. (2018) further demonstrated through eye-tracking that one month of continuous use of circularly polarized displays led to higher visual tracking gain and lower corrective saccade frequency compared with linear polarized displays. Mou et al. (2022) confirmed via eye-tracking and standardized questionnaires that circularly polarized smartphones significantly reduced dry eye symptoms and visual fatigue under prolonged use.

Although the existing researches have preliminarily revealed the influence of polarized light characteristics on visual fatigue, several limitations remain. First, most studies rely on blink rate or subjective ratings and lack comprehensive assessment of neurophysiological signals such as EEG (Monteiro et al., 2019; Tian et al., 2022). Second, insufficient control of external variables in experimental designs, including lighting and display parameters, limiting the elimination of confounding effects. Third, there is a paucity of research integrating subjective and objective assessment methods. Therefore, a more comprehensive evaluation framework is needed to fully assess the effects of light polarization on visual health, encompassing subjective scale, ocular behavior, and EEG measures.

In terms of methodology, this study integrated subjective and objective indicators. Subjective measures included a visual fatigue questionnaire and the Karolinska Sleepiness Scale (KSS). The visual fatigue questionnaire covers 13 symptom dimensions: blurred vision, dry eye, eye pain, eye tightness, eye irritation, eye burning, diplopia, tearing, headache, dizziness, increased blink rate, fatigue, and difficulty focusing (Sheedy et al., 2003). Items are rates on an 11-point Likert scale (0–10), with 0 indicating no symptom and 10 indicating severe symptom. The KSS assesses participants’ subjective alertness (Akerstedt and Gillberg, 1990), ranging from 1 (extremely alert) to 9 (extremely sleepy). Objective measures included blink rate and EEG characteristics. Blink rate reflects the activity level of blinking per unit time and typically increases under prolonged or high-visual-load tasks to maintain corneal moisture and visual comfort (Bafna and Hansen, 2021; Stern et al., 1994). Spectral changes in specific frequency of EEG are associated with fatigue: Theta and Alpha power increase as alertness and cognitive performance decline. Beta power decreases as the high-frequency activity of the cerebral cortex reduces, indicating a decline in attention, alertness and cognitive activation levels (Ibrahim et al., 2023; Trejo et al., 2015). The (θ + α)/β ratio is recognized as a key fatigue-related indicator (Jap et al., 2009; Peng et al., 2023; Xiaoli et al., 2016). Alpha centroid frequency reflects cortical rhythmic activity and information processing efficiency; it typically decreases under fatigue as spectral energy shifts toward lower frequencies (Jia, 2024; Ng and Raveendran, 2007). To comprehensively evaluate the effects of different polarized displays on visual fatigue, after Z-score standardization, this study used the entropy method to calculate a weighted composite index, integrating subjective visual fatigue, alertness, blink rate, (θ + α)/β ratio, and Alpha centroid frequency (Tian et al., 2022).

Based on this framework, the present study employed a randomized controlled within-subject design to compare circularly and linearly polarized displays under prolonged visual tasks. By analyzing multimodal data, we aim to elucidate the mechanisms through which light polarization affects the visual system and provide evidence to inform healthy display design and related ergonomic standards.

## Methods

### Participants

This study employed a within-subject design and determined the required sample size using G*Power software. The calculation was based on an effect size of f = 0.4, a significance level of α = 0.05, statistical power of 0.95, one group, four repeated-measures conditions (2 polarization types × 2 time points), resulting in a minimum required sample size of 15 participants (Faul et al., 2007). To further enhance the stability and representativeness of the results, 18 participants were actually recruited. Each participant completed two testing sessions scheduled on different days at the same time period to control circadian effects. All participants were right-handed, with normal or corrected-to-normal vision, no color blindness or color weakness, and no history of psychiatric disorders or family history of mental illness. The participants’ ages ranged from 20 to 38 years (mean = 26.89 ± 5.13), with an equal male-to-female proportion to ensure sample balance and representativeness. To ensure a consistent baseline state, participants underwent visual fatigue screening and completed standardized practice tasks before each experimental session, in order to reduce initial fatigue levels and increase familiarity with the tasks. Prior to the formal experiment, all participants read and signed the informed consent forms, clearly understanding the study purpose and procedures. The Ethics Committee of the University Committee on Human Research Protection at Tianjin Normal University approved this study.

### Display samples

The experimental stimuli consisted of two types of displays with different polarization characteristics: circularly polarized and linearly polarized. Except for their polarization states, the two displays were matched in other key optical parameters, including luminance, color temperature, and spectral distribution, ensuring the comparability of experimental conditions and the validity of the results. The displays used in this study were 27-inch LCD modules with IPS panels, a resolution of 1,920 × 1,080 pixels, and a typical brightness of 285 cd/m^2^. Luminance and color temperature were verified by using a luminance meter prior to the experiment. By controlling these factors, any observed differences in outcomes could be primarily attributed to the differences in polarization. Circular polarization is achieved by combining a linear polarizer and a quarter-wave plate. The linear polarizer first filters the incident light into a single polarization direction, and the quarter-wave plate then introduces a 90° phase delay between the orthogonal components of the light wave, converting it into circularly polarized light (Huard, 1997). The polarization characteristics of the screens were confirmed using a detector module. Specifically, the screen’s output was measured after passing through a polarizer, and the resulting brightness variations were recorded to determine whether the light was circularly or linearly polarized.

### Experimental task

The study employed a static Landolt C visual search task as the visual fatigue induction paradigm (Lindong and Ruifeng, 2016). The task lasted 40 min, gradually accumulating visual load under relatively stable visual operations, and inducing visual fatigue. Throughout the experiment, the illuminance, display luminance, and task parameters were kept consistent to minimize the external interference and ensure the reliability of the fatigue induction.

### Experimental procedure

The experiment was divided into four stages: preparation, a 3-min baseline period before the task, a 40-min main Landolt C visual task, and a 3-min baseline period after the task. Subjective visual fatigue questionnaires were administered both before and after the task, while EEG and VEOG data were continuously recorded throughout both the baseline and task periods.

### EEG and EOG data acquisition and processing

In this study, EEG signals were recorded using the Neuroscan SynAmps2 system, with the electrode placement following the international 10–20 system. EEG signals were collected from FP1, FP2, M1, and vertical electrooculography (VEOG) channels. The reference electrode (REF) was placed on the right mastoid (M2), and the ground electrode (GND) was positioned at FCz. Two electrodes of the VEOG channel were placed above (VEOU) and below (VEOL) the left eye to record vertical eye movement potentials. The sampling rate was set to 1,000 Hz to ensure adequate temporal and frequency resolution. All electrode impedances were maintained ≤5 kΩ.

Offline EEG preprocessing was performed using the EEGLAB toolbox (Delorme and Makeig, 2004) and the ERPLAB plugin (Lopez-Calderon and Luck, 2014). The main steps included: re-referencing EEG signals to the average of the signal of bilateral mastoids (M1 and M2); applying a 0.1–35 Hz band-pass filter to remove low-frequency drifts and high-frequency muscle artifacts; visually inspecting and rejecting abnormal segments; estimating and correcting blink-related VEOG artifacts using a linear regression model (Semlitsch et al., 1986); and extracting frequency-domain features via short-time Fourier transform (window = 3 s, overlap = 1 s) across Delta (1–4 Hz), Theta (4–8 Hz), Alpha (8–13 Hz), and Beta (13–30 Hz) bands (Cohen, 2014).

Offline VEOG data processing was conducted using the EEGLAB toolbox (Delorme and Makeig, 2004) and the EEG-Blinks plugin (Kleifges et al., 2017). Blink events were identified by analyzing characteristic waveforms in VEOG channels and frontal electrodes (FP1, FP2), combined with a multidimensional thresholding algorithm. Blink rate was then calculated for each participant.

### Statistical analysis

Statistical analyses were performed using JASP 0.18.3 (Jasp Team, 2023). Normality tests were first conducted for difference between each dependent variable. Parametric tests, such as paired *t*-tests or repeated-measures ANOVA, were used when data met normality assumptions; otherwise, non-parametric tests were applied. The independent variables were polarization type (linear vs. circular) and time stage (pre-task vs. post-task). Additionally, the entropy weight method was employed to determine the weight of each dependent variable, allowing for a weighted composite calculation of subjective and objective indicators (Yang, 2006).

## Results

### Subjective indicators

**FIGURE 1:**
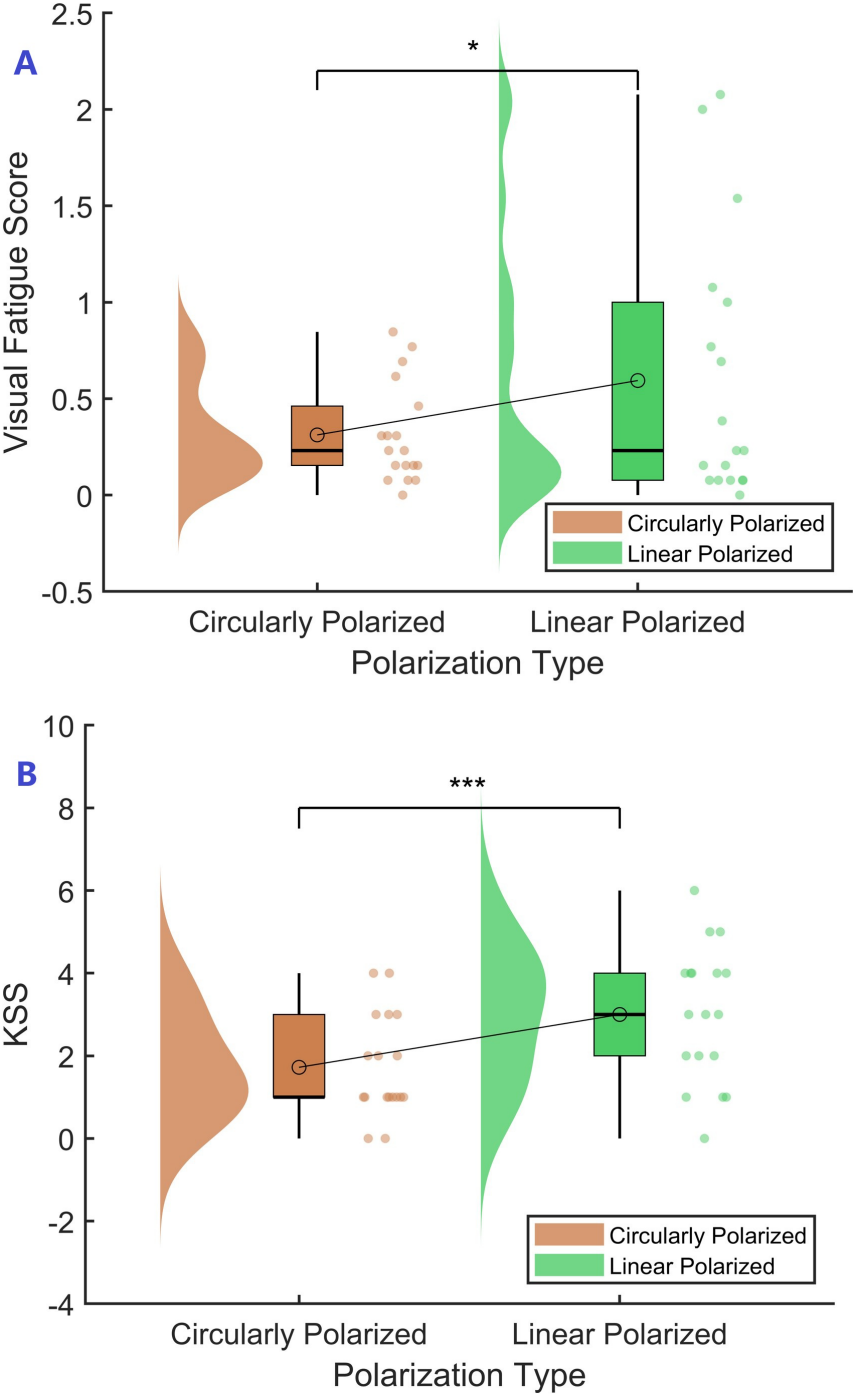
Visual An error in the conversion from LaTeX to XML has occurred here. 21 fatigue and Karolinska Sleepiness Scale (KSS) score for different polarized displays. **(A)** Subjective visual fatigue score. **(B)** Subjective alertness score. * Indicates a statistically significant difference at the 95% confidence level (*p* < 0.05), and *** indicates a statistically significant difference at the 99.9% confidence level (*p* < 0.001).

The visual fatigue score for each participant was calculated as the mean of the differences between the post-task and pre-task scores of all questionnaire items, serving as a quantitative indicator of overall subjective visual fatigue. Polarization type was treated as the independent variable, and the subjective visual fatigue score was the dependent variable. Normality tests indicated that the score differences for circularly polarized displays (0.31 ± 0.26) and linearly polarized displays (0.59 ± 0.68) were not normally distributed (Shapiro-Wilk test, *p* < 0.05). Therefore, the Wilcoxon signed-rank test was used for comparative analysis. Results showed that the subjective visual fatigue score was significantly lower on circularly polarized displays than on linearly polarized displays (*Z* = –2.045, *p* = 0.044, effect size = –0.667), suggesting that circular polarization has a significant advantage in mitigating subjective visual fatigue (See Figure 1A).

Each participant’s subjective alertness score was calculated as the difference between post-task and pre-task ratings. Polarization type was treated as the independent variable, and the subjective alertness score as the dependent variable. Normality tests indicated that the score differences for circularly polarized displays (1.72 ± 1.23) and linearly polarized displays (3.00 ± 1.65) were normally distributed (Shapiro-Wilk test, *p* > 0.05). Therefore, a paired-sample *t*-test was used for analysis. Results showed that subjective alertness scores were significantly lower on circularly polarized displays compared to linearly polarized displays (*p* < 0.001, Cohen’s d = –1.006), suggesting that circular polarization has a significant advantage in mitigating decreases in subjective alertness (See Figure 1B).

### Objective indicators

#### Blink rate

**FIGURE 2:**
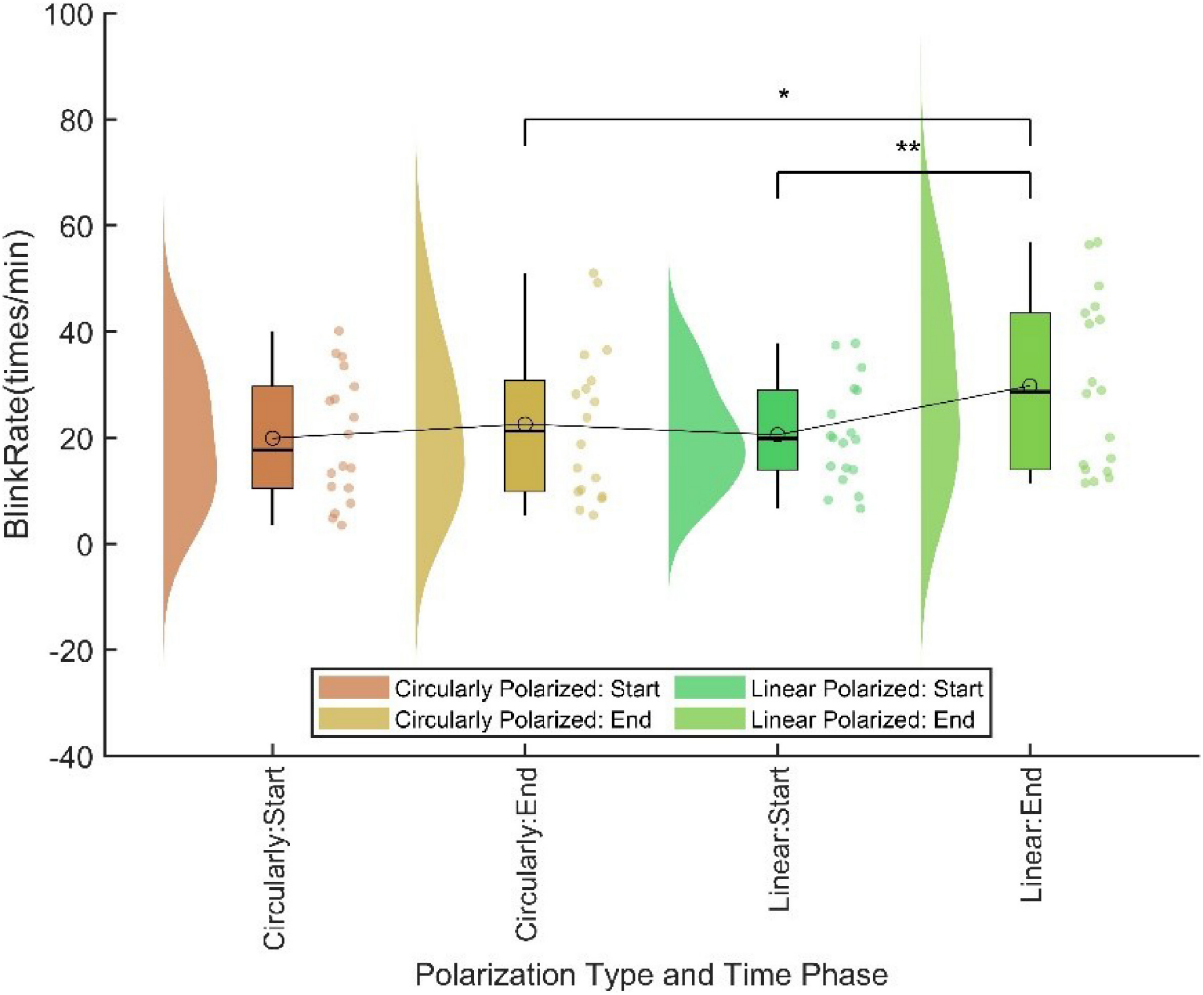
Blink rate at different time stages for displays with different polarization types. * Indicates a statistically significant difference at the 95% confidence level (*p* < 0.05), and ** indicates a statistically significant difference at the 99% confidence level (*p* < 0.01).

A two-way repeated measures ANOVA was conducted with polarization type (circular, linear) and time stage (beginning, end) as independent variables, and blink rate as the dependent variable. The results showed that the main effect of polarization was not significant (*F* = 3.597, *p* > 0.05), the main effect of time was significant (*F* = 7.334, *p* = 0.015), and the interaction effect between time stage and polarization type was significant (*F* = 5.932, *p* = 0.026). Given the significant interaction, simple effects analysis was further conducted, with Bonferroni correction applied for multiple comparisons (See Figure 2). The analysis revealed that for circular polarization, the difference between the beginning and end stages was not significant (*p* > 0.05, Cohen’s *d* = −0.199), whereas for linear polarization, the difference was significant (*p* = 0.008, Cohen’s *d* = −0.695). At the beginning stage, there was no significant difference between circular and linear polarization (*p* > 0.05, Cohen’s *d* = −0.048), but at the end stage, the difference was significant (*p* = 0.040, Cohen’s *d* = −0.544). These results indicated that after 40 min of continuous task performance, blink rate was significantly higher under linear polarization compared to circular polarization, suggesting that circularly polarized displays are more effective than linearly polarized displays in delaying ocular fatigue and maintaining visual comfort.

#### EEG

In this study, two EEG indicators—(*θ* + α)/*β* and alpha centroid frequency—were selected. These two indicators provide a more comprehensive evaluation of the neural correlates of visual discomfort and cognitive load. A higher (*θ* + α)/*β* value generally indicates that participants are in a relatively fatigued state, whereas a lower value corresponds to a more alert state. The alpha centroid frequency represents the weighted mean frequency within the alpha band and can be used to assess the stability and efficiency of cortical processing. A shift toward lower alpha frequency is typically associated with decreased attentional efficiency and reduced visual–cognitive performance.

**FIGURE 3:**
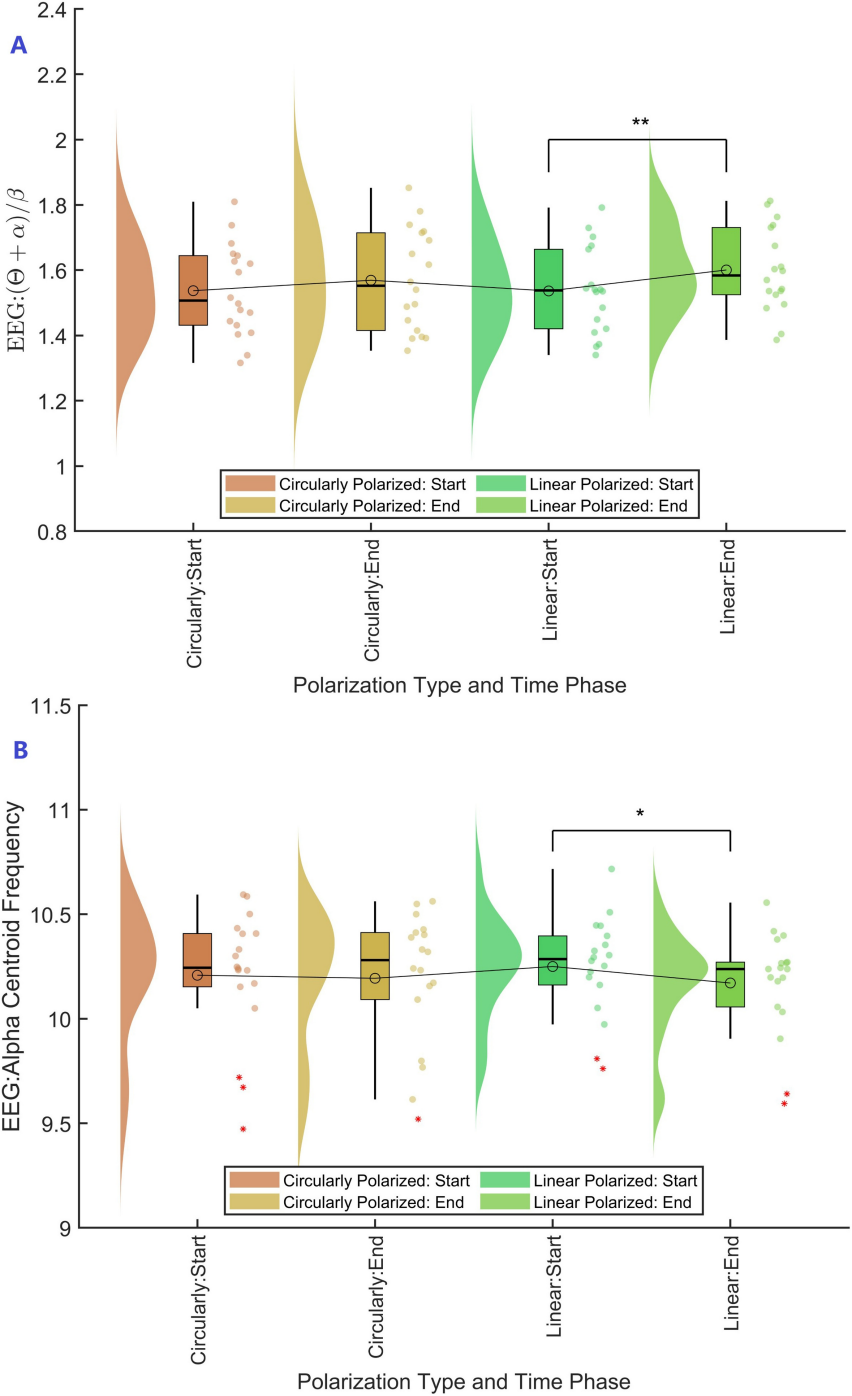
(θ + α)/β and alpha centroid frequency at different time stages for displays with different polarization types. * Indicates a statistically significant difference at the 95% confidence level (*p* < 0.05), and ** indicates a statistically significant difference at the 99% confidence level (*p* < 0.01).

With polarization type (circular, linear) and time stage (start, end) as independent variables and EEG (*θ* + α)/*β* ratio as the dependent variable, a two-way repeated measures ANOVA was conducted. The results showed that the main effect of polarization type was not significant (*F* = 0.915, *p* > 0.05), the main effect of time was significant (*F* = 13.319, *p* = 0.002), and the interaction effect between time and polarization type was not significant (*F* = 1.686, *p* > 0.05). Further simple effect analyses were performed, and Bonferroni correction was applied for multiple comparisons (See Figure 3A). The results indicated that for circular polarization, there was no significant difference between start and end stages (*p* > 0.05, Cohen’s *d* = −0.229), whereas for linear polarization, the difference between start and end stages was significant (*p* = 0.007, Cohen’s *d* = −0.460). Comparisons between circular and linear polarization at the start stage were not significant (*p* > 0.05, Cohen’s *d* = 0.005), nor at the end stage (*p* > 0.05, Cohen’s *d* = −0.226). These findings suggest that the (*θ* + α)/*β* ratio remained stable for circularly polarized displays, while for linear polarization, there was a significant increase after the task, indicating that long-duration exposure to linear polarization is more likely to induce EEG changes associated with visual fatigue. Although the difference between circular and linear polarization at the end stage was not statistically significant, circular polarization exhibited more stable EEG fatigue indicators throughout the task, demonstrating an advantage in alleviating visual fatigue.

With polarization type (circular, linear) and time stage (start, end) as independent variables and EEG Alpha centroid frequency as the dependent variable, a two-way repeated measures ANOVA was conducted. The results showed that the main effect of polarization type was not significant (*F* = 0.002, *p* > 0.05), the main effect of time was significant (*F* = 6.370, *p* = 0.022), and the interaction effect between time and polarization type was not significant (*F* = 2.809, *p* > 0.05). Further simple effect analyses were performed, and Bonferroni correction was applied for multiple comparisons (See Figure 3B). The results indicated that for circular polarization, there was no significant difference between start and end stages (*p* > 0.05, Cohen’s *d* = −0.242), whereas for linear polarization, the difference between start and end stages was significant (*p* = 0.041, Cohen’s *d* = −0.501). Comparisons between circular and linear polarization at the start stage were not significant (*p* > 0.05, Cohen’s *d* = 0.134), nor at the end stage (*p* > 0.05, Cohen’s *d* = −0.125). These findings suggested that under prolonged task conditions, the Alpha centroid frequency significantly decreased for linear polarization, reflecting an obvious tendency of neural activity slowing down, while for circular polarization, there was no significant fluctuation. This indicated that circular polarization had the potential to maintain the stability of visual-related EEG activity and reduce the risk of neural rhythm slowing during extended tasks.

### Composite evaluation

The statistical comparison of the composite fatigue levels across groups can integrate multidimensional data, thereby enhancing the accuracy and scientific rigor of visual fatigue assessment. The composite score (CE) is calculated as follows (See Equation 1).


$$C\,E=\sum_{i=1}^{5}S\,I\,n\,d\,e\,x_{i}*W_{i}$$ (1)

Where, CE represents the weighted subjective–objective composite score, with higher scores indicating greater fatigue; *SIndex*_*i*_ denotes the difference between post-task and pre-task values for the *i*-th indicator, normalized using min–max scaling. The Alpha centroid frequency is reverse-normalized to ensure directional consistency with other indicators; *W_i_* represents the weight of each indicator, determined via the entropy method, reflecting its relative importance in the composite score.

**FIGURE 4:**
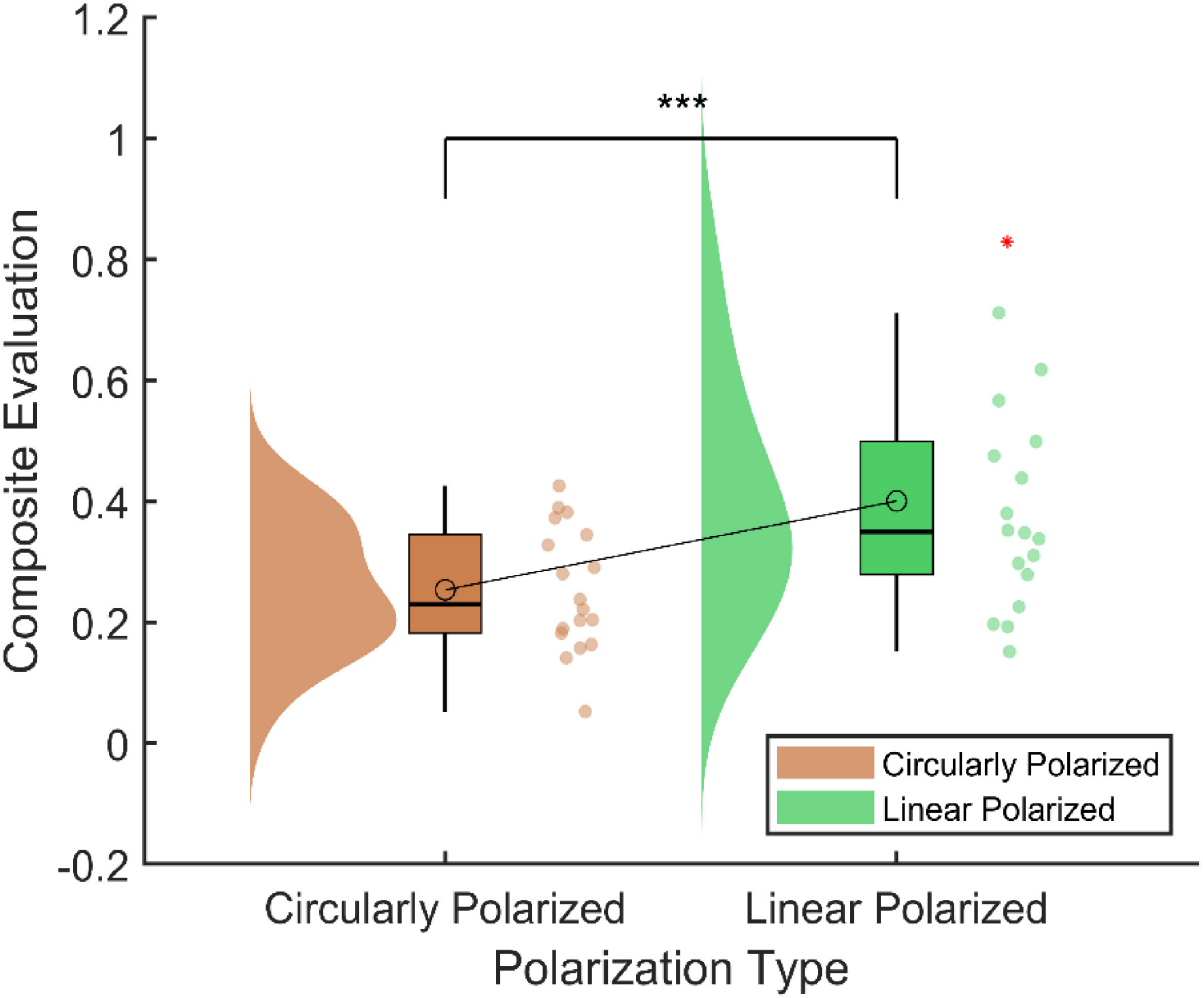
Composite evaluation for different polarized displays. *** Indicates a statistically significant difference at the 99.9% confidence level (*p* < 0.001).

Polarization type was used as the independent variable, and the weighted composite score as the dependent variable. Normality tests indicated that the score differences for circularly polarized (0.25 ± 0.10) and linearly polarized (0.40 ± 0.19) displays were normally distributed (Shapiro–Wilk test, *p* > 0.05). Therefore, a paired-sample *t*-test was conducted to examine differences. The composite evaluation revealed that circularly polarized displays significantly outperformed linearly polarized displays in alleviating visual fatigue. The composite score for circular polarization was significantly lower than that for linear polarization (*p* < 0.001, Cohen’s d = –1.069), indicating that circular polarization more effectively reduced subjective fatigue, maintains alertness, and stabilizes ocular and neurophysiological functions (See Figure 4). This multidimensional evaluation not only enhances the scientific accuracy of the assessment but also provides robust empirical support for optimizing display technologies and protecting visual health.

## Discussion

This study conducted a comprehensive analysis of circularly polarized and linearly polarized displays under prolonged visual task conditions from three dimensions: subjective experience, ocular behavior, and EEG physiology. The results consistently indicated that circular polarization had a significant advantage in alleviating visual fatigue.

In terms of subjective experience, visual fatigue and alertness scores under circularly polarized conditions were significantly better than those under linear polarization, which can be attributed to the similarity between circularly polarized light and natural light in the distribution of the electric field vector. Circular polarization, with its rotational symmetry, is not restricted to a single vibration direction, thereby avoiding visual adaptation burden caused by fixed polarization orientation and helping maintain visual comfort and attention levels during prolonged tasks (Mou et al., 2022).

Regarding ocular behavior, blink rate is an important indicator of visual fatigue and dry eye, closely related to the degree of visual discomfort. In this study, under linear polarization, participants’ blink rate increased significantly after the task and was notably higher than under circular polarization. Conversely, blink rate remained relatively stable under circular polarization. These findings are consistent with previous research and further support the role of circular polarization in reducing ocular surface dryness and improving visual comfort (Yin et al., 2022).

For EEG measures, the (*θ* + α)/*β* ratio and Alpha centroid frequency, respectively reflect low-frequency fatigue trends and cortical rhythm activity speed. Fatigue typically manifests as enhanced low-frequency activity, suppressed high-frequency activity and a downward shift of Alpha centroid frequency, indicating slowed neural rhythms and reduced processing efficiency. In this study, under linear polarization, participants exhibited a typical fatigue pattern after the task, with a significant increase in (*θ* + α)/*β* and a significant decrease in Alpha centroid frequency. In contrast, these indices remained relatively stable under circular polarization, suggesting its potential protective effect in maintaining cortical rhythm stability and cognitive processing efficiency. Although the group differences in EEG indices did not reach statistical significance, their trends were consistent with the subjective scale and ocular behavior results, providing cross-validation across multiple dimensions. In this study, EEG was recorded only from the frontal region to assess cognitive load and visual discomfort. Future studies will include occipital recordings to better capture visual cortex activity and further investigate the neural correlates of visual perception and fatigue.

Moreover, this study applied the entropy method to compute weighted composite scores for subjective visual fatigue, subjective alertness, blink rate, and EEG indices, establishing a multidimensional subjective–objective fatigue evaluation system. The weighted composite score analysis further confirmed the significant advantage of circularly polarized displays in alleviating visual fatigue, with composite scores substantially better than those of linear polarization. This multidimensional evaluation approach enhances the robustness of the findings and provides a systematic and objective tool for future research and display technology optimization.

## Conclusion

This study compared the effects of circularly polarized and linearly polarized displays on visual fatigue during prolonged visual tasks, and comprehensively analyzed changes in subjective visual fatigue, subjective alertness, blink rate, and two EEG indices—(*θ* + α)/*β* and Alpha centroid frequency. The results showed that after the task, circularly polarized displays produced significantly lower subjective visual fatigue scores and smaller declines in alertness compared with linear polarization. Regarding blink rate, circularly polarized displays showed no significant change between pre and post task, whereas under linear polarization, there was a significant increase, indicating greater ocular fatigue. For EEG indices, circular polarization maintained stable values in both (*θ* + α)/*β* and Alpha centroid frequency, while linear polarization showed a significant increase in (*θ* + α)/*β* and a significant decrease in Alpha centroid frequency, reflecting notable neural activity slowing. Overall, the circularly polarized displays are more effective in reducing subjective visual fatigue, maintaining alertness, alleviating ocular strain, and preserving EEG rhythm stability during prolonged visual tasks, demonstrating their advantage in mitigating visual fatigue.

## Data Availability

The datasets presented in this article are not readily available because the datasets generated and analyzed during the current study are available from the corresponding author upon reasonable request. Requests to access the datasets should be directed to YZ, zhangyh@cnis.ac.cn.
